# Demographic and clinical profile of adolescents suicide attempters admitted to an emergency department during the COVID-19 pandemic – a retrospective cohort study using hospital information system

**DOI:** 10.3389/fpsyt.2024.1422008

**Published:** 2024-06-17

**Authors:** Lucyna Tomaszek, Paulina Kurleto, Edyta Turkanik, Ewa Bielec, Irena Milaniak, Wioletta Dąbrowska-Mędrzycka

**Affiliations:** ^1^ Department of Specialist Nursing, Faculty of Medicine and Health Sciences, Andrzej Frycz Modrzewski Krakow University, Kraków, Poland; ^2^ Department of Thoracic Surgery, Institute of Tuberculosis and Lung Diseases, Rabka-Zdrój, Poland; ^3^ Department of Anaesthesiology and Intensive Care Nursing, Medical University of Gdansk, Gdansk, Poland

**Keywords:** suicide attempts, adolescents, COVID-19 pandemic, demographic emergency, emergency department

## Abstract

**Background:**

The prevalence of suicidal attempts among adolescents during COVID-19 significantly increased compared with pre-pandemic estimates. The aim of the study was to explore the demographic and clinical profile of adolescent suicide attempters admitted to the emergency department during the COVID-19 pandemic.

**Material and method:**

The retrospective analysis included, on the basis of electronic medical records in the CliniNet system, sociodemographic and clinical data of patients aged 10–18 years with a diagnosis of suicide attempt. Follow-up period: from March 20, 2020 to May 16, 2023.

**Results:**

During the COVID-19 pandemic, there were 425 emergency department visits among adolescents aged 11–17 due to a suicide attempt, with the largest number in the 15–17 (69%) age range. The percentage of emergency department visits was higher among females (80%) and urban residents (75.3%). Self-poisoning was the most common cause of suicide attempts (52.4%), followed by self-harm (41.4%), hanging (3.2%) and jumping from a height (2.1%). The most common toxic substances in self-poisonings were antidepressants and antipsychotics, followed by paracetamol. About 70% of visits were associated with adolescent mental disorders, of which depressive disorder was the most common. One death per 425 visits was recorded (0.2%).

**Conclusions:**

Adolescents attempting suicide during COVID-19 were most likely female, aged 15–17, city dwellers, undergoing psychiatric treatment mainly for depressive disorders. The mental health consequences of the pandemic may be more long term, and further monitoring will be needed in the years to come.

## Introduction

The COVID-19 pandemic, announced by the World Health Organization (WHO) on March 11, 2020 (1) was perceived both by adult and minors as a very stressful period (1, 2). Multiple factors were found to be associated with pandemic-related stress such as worrying about infection with the SARS-CoV-2 virus, the deterioration of a household’s economic status, social problems (e.g., difficult child-parent relationship, social isolation), school problems (e.g., experiencing difficulty completing schoolwork at home), or suboptimal physical environment (e.g., crowding at home) (3–5). Previous study has discovered a relationship between pandemic-related stressors and psychopathology—higher exposure to stressors was associated with increases in internalizing and externalizing psychopathology (3).

It was observed that the prevalence of mental health problems in adolescents during COVID-19 significantly increased compared with pre-pandemic estimates (4, 6). Racine et al. found, based on a meta-analysis of 29 studies involving 80,879 participants in the age 18 years or younger—from Europe, East Asia, North/Central/South America, and from the Middle East—that the incidence of depression and anxiety symptoms during the pandemic doubled compared to the pre-pandemic period (7).

The overall pattern of results, reported in previous study, also suggests that mental health problems—among them: depression, psychosis, anxiety/personality/eating disorders, trauma—are a general risk factors for suicide attempts (8). The method most frequently used by adolescents admitted to an emergency department due to a suicide attempt was exogenous intoxication, mainly by medication (9, 10). A systematic review and meta-analysis conducted by Madigan et al. provide good evidence of an increase in emergency department visits for attempted suicide during the COVID-19 pandemic. It is worth adding that analyzed sample included 11.1 million pediatric emergency department visits across 18 countries (11). Yard et al. noted that during the pandemic period, emergency department visits for suspected suicide attempts began to increase among adolescents aged 12–17 years (12).

Polish police data for the years 2012–2021 indicates a growing trend of suicidal behavior among people under 18 years of age. However, the sharp increase in the number of suicide attempts and suicide deaths in 2021 compared to 2020 (an increase of 77% in suicide attempts and 19% in suicidal deaths) or to the earlier period appears to be an adverse effect of the COVID-19 pandemic. It is also worth mentioning that in 2021 the number of girls who attempted suicide more than doubled compared to 2020 (1,086 vs. 538) (13). The results of other studies also suggest that the increase in suicidal attempts are more conclusive for girls than for boys (14–16).

The COVID-19 pandemic is associated with deterioration of young people’s mental health, the consequences of which may be long-term and further monitoring of their health will be necessary in the coming years. Additionally, knowing the profile of adolescents admitted to the emergency department due to a suicide attempt may allow the ability to develop targeted, holistic suicide prevention strategies and strengthen the role of the emergency departments in identifying the special clinical needs of these patients. Thus, the aim of the study was to explore the demographic and clinical profile of adolescent suicide attempters admitted to the emergency department during the COVID-19 pandemic.

## Materials and methods

### Study design, setting, and ethical consideration

This was a retrospective cohort study. We analyzed 425 electronic health records of patients aged 10–18 years, admitted to the Emergency Department of the University Children’s Hospital in Krakow due to suicide attempts between March 20, 2020 and May 16, 2023 (the state of epidemic announced by the Polish Minister of Health in connection with SARS-CoV-2 virus infections – in this study called ‘the COVID-19 pandemic) (17). The study followed the guidelines of RECORD statement (The Reporting of studies Conducted using Observational Routinely collected health Data) (18). This study was conducted after obtaining the consent of the Bioethics Committee of the Andrzej Frycz Modrzewski Krakow University (opinion nr KBKA/53/O/2022). The obtaining data from the medical records were completely anonymous.

### Participants

**Figure 1:**
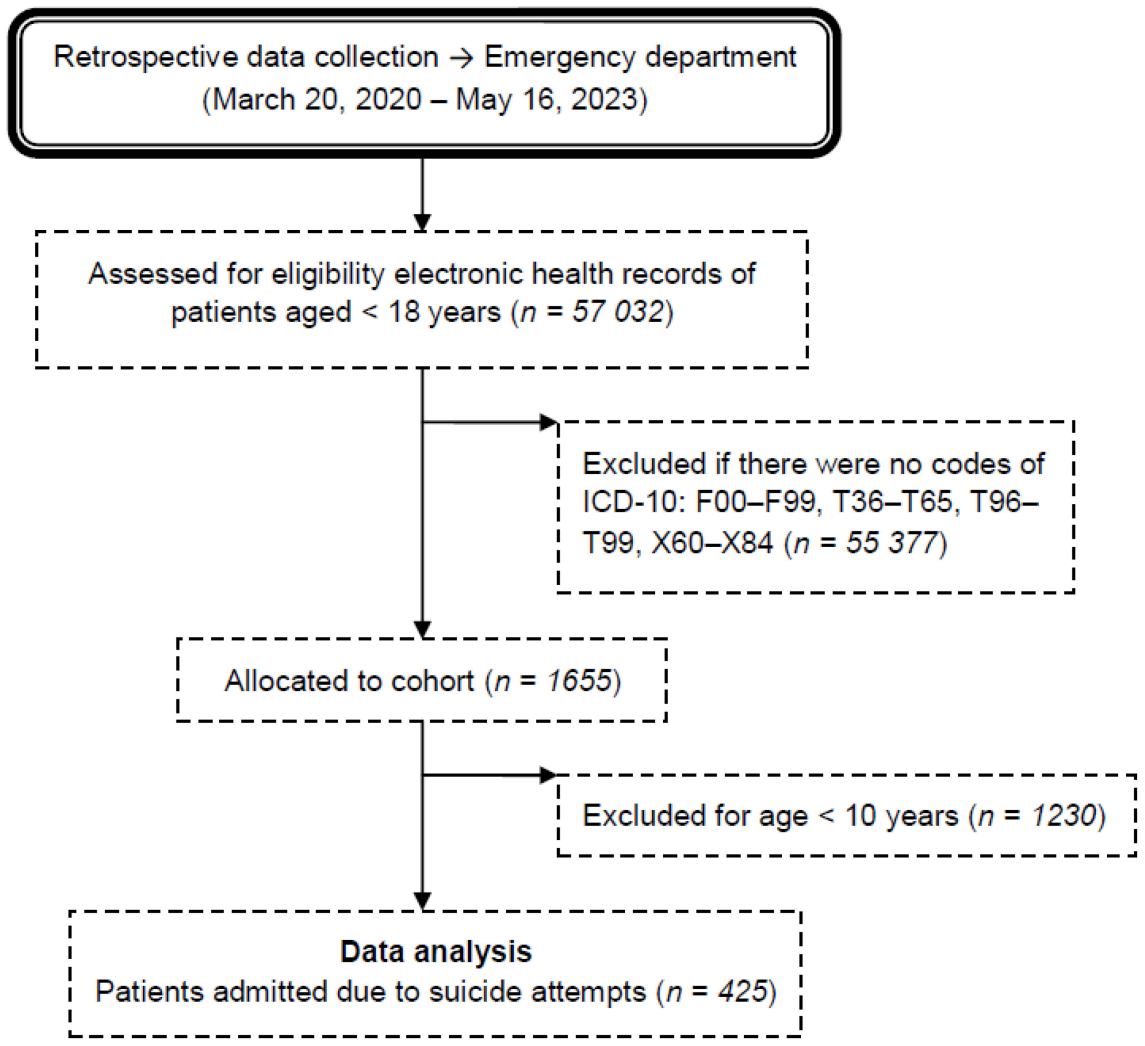
Flow chart of the medical record review process for patients admitted to the emergency department due to suicide attempts during the COVID-19 pandemic. ICD-10 → International Statistical Classification of Diseases and Related Health Problems 10th Revision. F00–F99 → Mental and behavioral disorders. T36–T50 → Poisoning by drugs, medicaments and biological substances. T51–T65 → Toxic effects of substances chiefly nonmedicinal as to source. T96 → Sequelae of poisoning by drugs, medicaments and biological substances. T97 → Sequelae of toxic effects of substances chiefly nonmedicinal as to source. T98 → Sequelae of other and unspecified effects of external causes.X60–X84 →Intentional self-harm. Incl.: purposely self-inflicted poisoning or injury suicide (attempted).

All patients diagnosed with suicidal attempts, both sexes, aged between 10–18 years, were included (**Figure 1**). Eligible records were emergency department billing records with an ICD-10-CM code: F00–F99 (Mental and behavioral disorders), T36–T50 (Poisoning by drugs, medicaments and biological substances), T51–T65 (Toxic effects of substances chiefly nonmedicinal as to source), T96 (Sequelae of poisoning by drugs, medicaments and biological substances), T97 (Sequelae of toxic effects of substances chiefly nonmedicinal as to source), T98 (Sequelae of other and unspecified effects of external causes), and X60–X84 (Intentional self-harm. Incl.: purposely self-inflicted poisoning or injury suicide [attempted]) (19).

Duplicates of electronic health records and those whose data was not fully completed were excluded.

### Variables

Data regarding people trying to commit suicide included: age, gender, place of residence and date of the suicide attempt, frequency, methods and type of toxic substance used during the suicide attempt, existing mental and behavioral disorders, psychiatric treatment, health condition of respondents after a suicide attempt, as well as the type of medical interventions undertaken. Data sources were: administrative billing records for emergency department visits and electronic medical records in the CliniNet system.

### Statistical analysis

Data were analyzed using STATISTICA v.13 (TIBCO Software Inc., Kraków, Poland 2017). Qualitative data were presented as numbers and percentages. Continuous data are presented as median (percentiles 25–75). Relationships between qualitative variables determined using the chi-square test or Fisher’s exact test. The Mann-Whitney test was used for quantitative comparison. The normality of distribution was tested using the Shapiro-Wilk test. A p value < 0.05 was considered significant.

## Results

### Frequency of suicide attempts depending on age, gender, and place of residence

**Figure 2:**
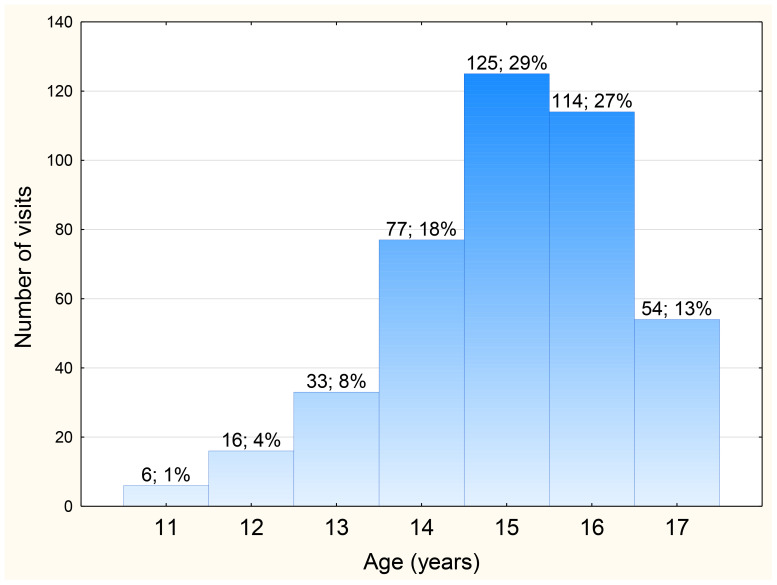
Distribution of the number of emergency department visits among adolescents due to suicide attempts during the COVID-19 pandemic depending on age.

**Figure 3:**
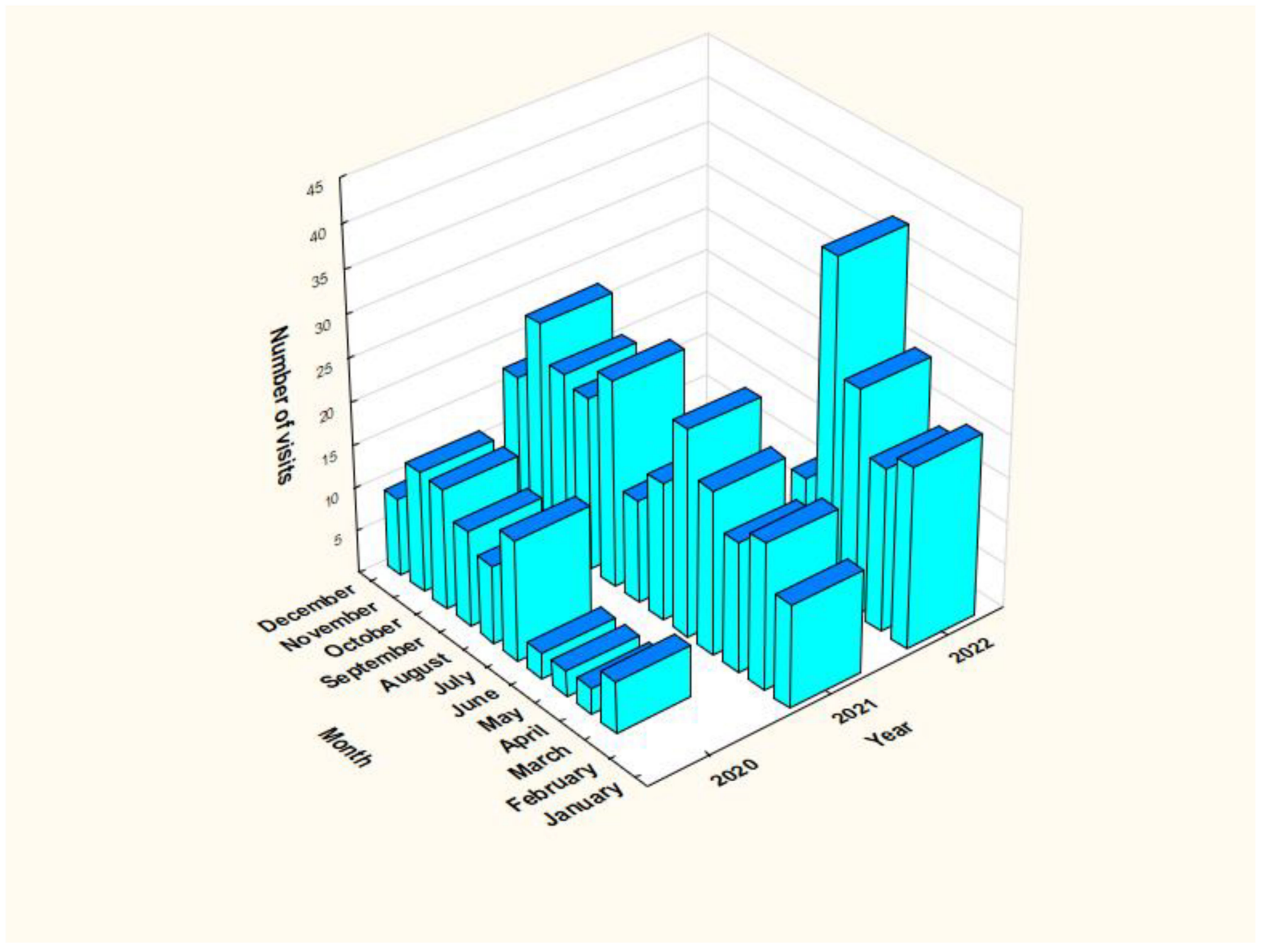
Distribution of the number of emergency department visits among adolescents due to suicide attempts during the COVID-19 pandemic depending on the month and year (from March 20, 2020 to May 16, 2022).

During the study period, there were 425 emergency department visits due to suicide attempts among adolescents aged 10–18 years (**Table 1**; **Figure 2**). The most suicide attempts occurred among girls (79.8%) and urban residents (75.3%). Suicide attempts remained more common among minors aged 15–17 (68.9%) than at the age 11–14 (31.1%). Gender distribution (χ2 = 1.51; p = 0.22) and place of residence (χ2 = 1.11; p = 0.73) in both age groups were similar. Every third visit concerned a patient who made more than one suicide attempt during the studied period. It was observed that the longer the pandemic lasted, the higher the number of suicide attempts (**Figure 3**).

**Table 1:** Characteristics of emergency department visits among adolescents due to suicide attempts during the COVID-19 according to sex, age and place of residence.

Variables	Number of emergency department visits (%)
Number of suicide attempts	
** • One**	284 (66.8)
** • ≥ Two**	141 (33.2)*
Sex	
** • Female**	339 (79.8)
** • Male**	86 (20.2)
Age (years)	
** • 11–14**	132 (31.1)
** • 15–17**	293 (68.9)
Residence	
** • Urban**	320 (75.3)
** • Rural**	105 (24.7)

*Urban residents were more likely to make at least two suicide attempts than rural residents (37.2% vs. 20.9%; χ2 = 9.39; p = 0.002).

### Suicide method

**Figure 4:**
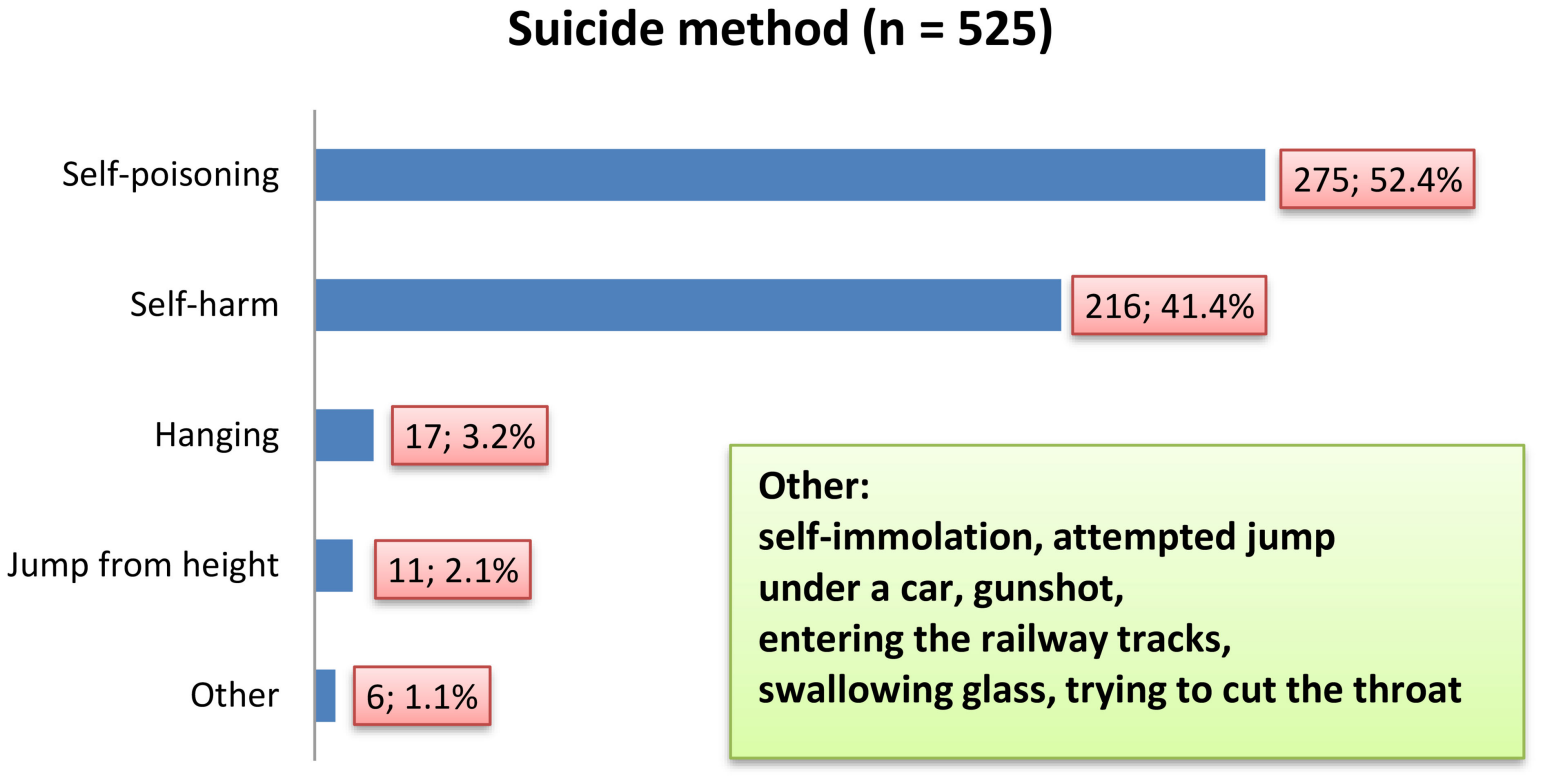
Characteristics of emergency department visits among adolescents during the COVID-19 according to suicide method.

The suicide attempt methods chosen by teenagers are presented on **Figure 4**. Self-poisoning was most often attempted by teenagers, both in the age group over 14 and younger (n = 85; 64.8% vs. n = 190; 64.9%; χ2 = 0.0001; p = 0.99) and in the group of girls and boys (n = 225; 66.6% vs. n = 50; 58.1%; χ2 = 2.14; p = 0.14). These differences were not statistically significant. Self-poisoning, as a form of suicide, was more often chosen by teenagers living in the countryside than in the city (n = 77; 74% vs. n = 198; 61.9%; χ2 = 5.09; p = 0.02). Self-harm was associated primarily with female gender (n = 181; 53.4% vs. n = 35; 40.7%; χ2 = 4.42; p = 0.03), while hanging (n = 7; 8.1% vs. n = 10; 2.1%; χ2 = 4.81; p = 0.028), and jump from height (n = 7; 8.1% vs. n = 4; 1.2%; χ2 = 13.2%; p = 0.0003) with male gender.

Every fourth emergency department visit (n = 97; 22.9%) was the result of teenagers using two methods to kill themselves – the most common were self-poisoning and self-harm (n = 86; 20.2%). It should be mentioned that three suicide attempt methods were registered in the age group over 14 among two boys and one girl. The percentage of emergency department visits caused by the use of more than one suicide method was similar both in the age group over 14 and younger (n = 63; 21.5% vs. n = 34; 25.8%; χ2 = 0.93; p = 0.33), in the group of girls and boys (n = 82; 24.2% vs. n = 15; 17.4%; χ2 = 1.77; p = 0.18), and minors living in rural areas and cities (n = 29; 27.6% vs. n = 68; 21.2%; χ2 = 1.82; p = 0.17).

### Type of toxic substance

Self-poisoning was the cause of 275 emergency department visits, mainly due to the consumption of drugs (n = 227; 82.5%). In this group, 8.3% (n = 23) of visits involved a combination of drugs and alcohol, while approximately 8% (n = 21) of visits were caused by the use of alcohol alone.

Girls chose drugs for suicide purposes more often than boys (n = 209; 61.6% vs. n = 41; 47.7%; χ2 = 5.53; p = 0.02), and rural residents (n = 72; 68.6% vs. n = 178; 55.6%; χ2 = 5.47; p = 0.02). Alcohol was consumed more often by boys than girls (n = 14; 16.3% vs. n = 39; 8.8%; χ2 = 4.08; p = 0.04), and minors over 14 years of age consumed it more than twice as often as in the younger group (n = 37; 12.6% vs. n = 7; 5.3%; χ2 = 5.26; p = 0.02). Among all drugs used for self-poisoning (**Table 2**), both in single-drug and multi-drug poisonings, the largest percentage were antidepressants and antipsychotics, followed by paracetamol. The percentage of emergency department visits due to self-poisoning with antidepressants and antipsychotics (n = 91; 31.3% vs. n = 22; 16.4%; χ2 = 1037; p = 0.001) and hypnotics/sedatives/anticonvulsants (n = 28; 9.6% vs. n = 4; 3.0%; χ2 = 5.80; p = 0.02) was higher among adolescents receiving psychiatric treatment than among those not treated. The opposite situation occurred in the case of the use of paracetamol (n = 32; 23.9% vs. n = 39; 13.4%; χ2 = 7.23; p = 0.007) and other non-opioid analgesics (n = 24; 17.9% vs. n = 23; 7.9%; χ2 = 9.34; p = 0.002). Moreover, paracetamol was more often used by girls (n = 63; 18.6% vs. n = 8; 9.3%; χ2 = 4.25; p = 0.04) and rural residents (n = 27; 25.7% vs. n = 44; 13.7%; χ2 = 8.13; p = 0.004).

**Table 2:** Drugs used in deliberate self-poisoning among adolescents admitted to the emergency department during the COVID-19 pandemic.

Drugs	Number (percentage)		
	Overall n = 330 (100)	Single-drug n = 185 (100)	Multiple-drug n = 145 (100)
Antidepressants and antipsychotics	113 (34.2)	81 (43.8)	32 (22.1)
Hypnotics and sedatives, anxiolytics, antiepileptics	32 (9.7)	12 (6.5)	20 (13.8)
Paracetamol	71 (21.5)	40 (21.6)	31 (21.4)
Other analgesics	47 (14.2)	18 (9.7)	29 (20)
Cardiovascular drugs	8 (2.4)	4 (4.3)	4 (2.7)

Single-drug and multi-drug poisonings occurred with similar frequency among girls and boys (χ2 = 0.27; p = 0.60), adolescents living in rural areas and cities (χ2 = 0.30; p = 0.58), and in the age group both over 14 and younger (χ2 = 0.46; p = 0.50).

### A type of mental disorder

The study showed that suicide attempts were often associated with mental disorders. Depression was the most common cause of emergency department visits (n = 187; 44%), mainly among patients aged 15–16 years (n = 71). Girls diagnosed with depression have visited the emergency department more often than boys (n = 160; 47.2% vs. n = 27; 31.4%; χ2 = 6.95, p = 0.008). However, eating disorders were exclusively associated with the female gender (n = 16, 3.7%) – the youngest patient was 12 years old, and the most emergency department visits for this reason were among 16-year-old patients (n = 7). Emotion disorders (n = 18; 20.9% vs. n = 37; 10.9%; χ2 = 6.12, p = 0.013) and personality disorders (n = 7; 8.14% vs. 8; 2.4%; χ2 = 6.73; p = 0.009) were more predominated among boys.

### Suicide attempts depending on the type of duty

55.5% (n = 236) of all emergency department visits due to suicide attempt occurred during night shifts. During this time, people with suicidal intentions after drinking alcohol were admitted more often than during the day duty (n = 36; 15.2% vs. n = 8; 4.2%; χ2 = 13.73; p = 0.0002). However, the day-time emergency department was more frequently visited by adolescents who had attempted suicide by hanging (n = 12; 6.3% vs. n = 5; 2.1%; χ2 = 4.89; p = 0.026), with adjustment disorders (n = 11; 5.8% vs. n = 4; 1.7%; χ2 = 5.24; p = 0.02), and nutrition disorders (n = 11; 5.8% vs. n = 5; 2.1%; χ2 = 3.97; p = 0.046).

A total of 215 medical services were provided in the emergency department in the form of dressing wounds after self-harm (50.6% of emergency department visits). Almost every sixth patient required gastric lavage and administration of activated charcoal via a gavage (n = 71; 16.7% of hospitalizations), and every tenth patient needed an antidote (n = 43; 10.1%). In 5.2% of cases (n = 22), patients required treatment in the pediatric intensive care unit. One fatal case per 425 emergency department visits was recorded (0.2%).

## Discussion

The study showed that adolescents attempting suicide during COVID-19 pandemic are mostly female, aged 15–17, city dwellers, undergoing psychiatric treatment for depressive disorders. The most common method of suicide attempts were deliberate self-poisonings with prescription drugs followed by over-the-counter analgesics such as paracetamol.

This study revealed, that during the period of the COVID-19 pandemic, girls visited the emergency department for suicide attempts more often than boys (79.8% vs. 20.2%). These findings are consistent with the results of another Polish study conducted by Pilarska et al. in the age group of 8–17 years, where approximately 80% of this group were girls (10). Similar results were found by Kirič et al. (14) among the Slovak children and adolescents, who required emergency help with suicidality and attempted suicide. They assessed suicidal behavior more frequent among females than males (69.3% vs. 30.7). Gender disparity in suicide attempts in community adolescents and young adults aged 12–26 years was confirmed by Miranda-Mendizabal et al. (8) in their systematic review and meta-analysis of longitudinal studies. They found that females had an almost twice higher risk of suicide attempts than males. The female preponderance in a suicidal tendency may be partly explained by greater risk of developing depressive or eating disorders (8, 20). Other risk factors specific to the female gender, but not assessed in our study, include: post-traumatic stress disorder, being a victim of dating violence, interpersonal problems and a previous abortion (8).

Our findings showed, that suicide attempts were significantly more common among adolescents aged 15–17 years than younger, and those living in urban than rural areas. Similar observations were obtained in the study by Zygo et al. (21), who in pre-pandemic period determined that more Polish minors attending schools in Eastern Poland, aged 17–18 reported suicidal thoughts, plans and attempts than those aged 13–16 years; most of them were city residents. Goldman-Mellor et al. (22) found, that before the pandemic, rural U.S. youth were significantly less likely to report suicide attempts than their urban counterparts. Salt et al. (23) identified any change in the incidence of suicidal attempts within any demographic subgroup of U.S. pediatric sample in the 6 months post‐SARS‐CoV‐2 when compared to the 6 months of the prior year, most likely due to group size limitations. However, they found an increase in incidence of suicidal ideation among adolescents aged 14–17, and those residing rural areas.

Both our studies and those conducted by others (24, 25) found an association between suicide attempt and depression. There is scientific evidence, that people who have experienced their first depressive episode (26) or depressive symptoms associated with the psychotic episode, in the early stages of the disorder, before the first admission or contact with health services (27), have received psychiatric treatment and have undergone psychotherapy in the past (28), are at high risk of attempting suicide. Furthermore, Ong et al. (25) showed, that depressive disorder among pediatric patients were associated with a higher likelihood of suicide attempt than attention deficit disorder, disruptive behavior disorder, bipolar disorder, or schizophrenia.

Based on the January 2020 and June 2021 date (n = 154) collected by Pilarska et al. (8), self-poisoning, followed by self-injury and jumping from height, emerged as the most common method of Polish pediatric patients who have attempted suicide. A similar order of occurrence of suicide methods was recorded in our own study. The main cause of self-poisoning, as in the study by Barbeito et al., was an overdose of prescribed drugs (27), such as antidepressants and antipsychotics, followed by paracetamol. Koppen et al. (29) found significant increase in deliberate self-poisonings during COVID-19 pandemic, with a preference for paracetamol, especially among 13-, 14-, and 15-year-old Dutch female adolescents. However, self-poisoning is less likely to result in death than hanging, shooting a firearm or jumping from a height (30).

Youth suicide rates increase with age, and females are less likely to die by suicide than males. This hypothesis was confirmed by Glenn et al. in a meta-analytic review of worldwide suicide rates among adolescents. They found that hanging/suffocation was the most common method of suicide across all countries and for both sexes (31). Pikala and Burzyńska who assessed mortality trends due to suicide in Poland in the years 2000–2019, reported higher percentage of suicide deaths in age 15–24 than 5–14 (10.2% vs. 0,27%). The percentage of fatal cases among girls in mentioned age groups was 8.4% and 0.6%, respectively (32).

In our study, we recorded one death per 425 emergency department visits (0.2%) – it was a 16-year-old girl who suffered sudden cardiac arrest as a result of hanging. In 5.2% of the cases, patients required treatment in the intensive care unit. Bruns et al. found that although among German adolescents serious suicide attempts requiring intensive care treatment dramatically increased during COVID-19 pandemic, fatal suicide rates remained stable (33).

To sum up, suicide attempts among adolescents are a serious and complex public health problem, that is constantly increasing and an exceptional time that may have aggravated this problem was the period of the COVID-19 pandemic. In Poland, the psychiatric and psychological care system does not meet the rapidly growing needs of children and adolescents in crisis. It should also be added, that in the last few years, Poland has taken the inglorious second place in the European Union in terms of suicide attempts among people under 18 years of age. This situation may be caused by many factors, including the fact that in Poland access to professional help, i.e. to psychologists and educators from whom young people could obtain professional help is still difficult. According to the Central Register of Doctors of the Republic of Poland kept by the Supreme Medical Council, as of March 31, 2024, there were 553 professionally active doctors with specialization in child and adolescent psychiatry registered in Poland. In accordance with the WHO recommendations, there should be 10 psychiatrists per 100,000 children. Although currently in Poland this ratio is 10.49, the waiting time for a visit is more than 30 days (34, 35) Currently, Poland is undergoing a reform of the psychiatric and psychological care system for children and adolescents. Within this framework, it is crucial to implement the first of three levels of reference, the task of which is to provide care in the patient’s environment (i.e. at home - in the family, at school – among peers) by psychologists, psychotherapists and community therapists (36).

## Limitation

Our study had several limitations. Firstly, the data was collected only in one of the available emergency departments for children and adolescents in the country, thus this data is not nationally representative. Secondly, because our study was a retrospective study, we were unable to assess and control many of the factors influencing suicidal behavior among adolescents. Thirdly, no pre-COVID-19 data had been collected for this specific population, which prevents the study from being able to assess whether and how the situation might have changed over the past year.

## Conclusion

Adolescents attempting suicide during COVID-19 pandemic were most often females, aged 15–17, city dwellers, undergoing psychiatric treatment mainly for depressive disorders. This study highlights the need to improve suicide prevention strategies among adolescents.

### Implications for clinical practice and future directions

Activities to create effective, comprehensive suicide prevention programs among young people, taking into account risk factors (e.g. females, aged 15–17) should be intensified. Among the preventive measures, early diagnosis of mental disorders (including depression), should be strengthened through appropriate public education and access to psychologists and psychiatric treatment. Such education and policies should also take into account the key role that social networks and community engagement play in promoting positive youth development, emotional support, help and access to resources in coping with such challenges (37). National information campaigns promoting ways to seek professional help in the event of a mental health crisis should also be more present. It is important that people after a suicide attempt receive continuous, individualized and coordinated psychiatric care. Future research should focus on longitudinal studies to monitor the long-term impact of the pandemic on mental health among adolescents, including suicidal behavior. Additionally, exploring protective factors and resilience-building strategies among adolescents could increase the effectiveness of preventive and intervention efforts (38).

## Data availability statement

The original contributions presented in the study are included in the article/supplementary material. Further inquiries can be directed to the corresponding author.

## Ethics statement

This study was conducted after obtaining the consent of the Bioethics Committee of the Andrzej Frycz Modrzewski Krakow University (opinion nr KBKA/53/O/2022). The obtaining data from the medical records were completely anonymous.

## Author contributions

LT: Writing – original draft, Project administration, Methodology, Investigation, Data curation, Conceptualization, Funding acquisition, Formal analysis, Visualization. PK: Writing – original draft, Data curation. ET: Writing – original draft, Methodology, Investigation. EB: Writing – review & editing, Data curation, Conceptualization, Investigation. IM: Writing – original draft, Investigation. WD-M: Writing – review & editing, Visualization, Supervision, Funding acquisition.

## References

[1] Cucinotta D Vanelli M. WHO declares COVID-19 a pandemic. Acta Biomed. (2020) 91:157–60. doi: 10.23750/abm.v91i1.9397 PMC7569573 32191675

[2] Juszczyk A Dragan M Grajewski P Holas P. Prevalence of adjustment disorder in Poland during the COVID-19 pandemic and its association with symptoms of anxiety and depression. Adv Psychiatry Neurol. (2021) 30:141–53. doi: 10.5114/ppn.2021.110764 PMC9881629 37082764

[3] Rosen ML Rodman AM Kasparek SW Mayes M Freeman MM Lengua LJ. Promoting youth mental health during the COVID-19 pandemic: A longitudinal study. PloS One. (2021) 16:e0255294. doi: 10.1371/journal.pone.0255294 34379656 PMC8357139

[4] Kang S Jeong Y Park EH Hwang SS. The impact of household economic deterioration caused by the COVID-19 pandemic and socioeconomic status on suicidal behaviors in adolescents: A cross-sectional study using 2020 korea youth risk behavior web-based survey data. J Prev Med Public Health. (2022) 55:455–63. doi: 10.3961/jpmph.22.241 PMC9561140 36229908

[5] Sikorska I Lipp N Wróbel P Wyra M. Adolescent mental health and activities in the period of social isolation caused by the COVID-19 pandemic. Adv Psychiatry Neurol. (2021) 30:79–95. doi: 10.5114/ppn.2021.108472 PMC9881619 37082432

[6] Agostino H Burstein B Moubayed D Taddeo D Grady R Vyver E. Trends in the incidence of new-onset anorexia nervosa and atypical anorexia nervosa among youth during the COVID-19 pandemic in Canada. JAMA Netw Open. (2021) 4:e2137395. doi: 10.1001/jamanetworkopen.2021.37395 34874405 PMC8652595

[7] Racine N McArthur BA Cooke JE Eirich R Zhu J Madigan S. Global prevalence of depressive and anxiety symptoms in children and adolescents during COVID-19: A meta-analysis. JAMA Pediatr. (2021) 175:1142–50. doi: 10.1001/jamapediatrics.2021.2482 PMC8353576 34369987

[8] Miranda-Mendizabal A Castellví P Parés-Badell O Alayo I Almenara J Alonso I. Gender differences in suicidal behavior in adolescents and young adults: systematic review and meta-analysis of longitudinal studies. Int J Public Health. (2019) 64:265–83. doi: 10.1007/s00038-018-1196-1 PMC6439147 30635683

[9] Fogaça VD Souza DM Silva L Guedes DMB Domingues F Trinquinato I. Suicide attempts by adolescents assisted in an emergency department: a cross-sectional study. Rev Bras Enferm. (2023) 76:e20220137. doi: 10.1590/0034-7167-2022-0137 37042925 PMC10084778

[10] Pilarska I Grabska K Stachurski J. Suicide attempts among children and adolescents admitted to a Polish Emergency Department: Analysis of epidemiology, circumstances and methods of 154 cases. Adv Clin Exp Med. (2023) 32:1377–84. doi: 10.17219/acem/162245 37140017

[11] Madigan S Korczak DJ Vaillancourt T Racine N Hopkins WG Pador P. Comparison of paediatric emergency department visits for attempted suicide, self-harm, and suicidal ideation before and during the COVID-19 pandemic: a systematic review and meta-analysis. Lancet Psychiatry. (2023) 10:342–51. doi: 10.1016/S2215-0366(23)00036-6 PMC10097509 36907199

[12] Yard E Radhakrishnan L Ballesteros MF Sheppard M Gates A Stein Z. Emergency department visits for suspected suicide attempts among persons aged 12–25 years before and during the COVID-19 pandemic - United States, january 2019-may 2021. MMWR Morb Mortal Wkly Rep. (2021) 70:888–94. doi: 10.15585/mmwr.mm7024e1 PMC8220953 34138833

[13] Suicide attempts among children and teens rose 77% in Poland last year (2022). Available online at: https://notesfromPoland.com/2022/02/11/suicide-attempts-among-children-rose-77-in-Poland-last-year/.

[14] Kirič B Leben Novak L Lušicky P Drobnič Radobuljac M. Suicidal behavior in emergency child and adolescent psychiatric service users before and during the 16 months of the COVID-19 pandemic. Front Psychiatry. (2022) 13:893040. doi: 10.3389/fpsyt.2022.893040 35633784 PMC9130484

[15] Gracia R Pamias M Mortier P Alonso J Pérez V Palao D. Is the COVID-19 pandemic a risk factor for suicide attempts in adolescent girls? J Affect Disord. (2021) 292:139–41. doi: 10.1016/j.jad.2021.05.044 PMC8777066 34119869

[16] Auger N Low N Chadi N Israël M Steiger H Lewin A. Suicide attempts in children aged 10–14 years during the first year of the COVID-19 pandemic. J Adolesc Health. (2023) 72:899e905. doi: 10.1016/j.jadohealth.2023.01.019 36870902 PMC9980433

[17] Rozporządzenie Ministra Zdrowia z dnia 20 marca 2020 r. w sprawie ogłoszenia na obszarze Rzeczypospolitej Polskiej stanu epidemii (2022). Available online at: https://isap.sejm.gov.pl/isap.nsf/DocDetails.xsp?id=WDU20200000491.

[18] Benchimol EI Smeeth L Guttmann A Harron K Moher D Petersen I. The REporting of studies Conducted using Observational Routinely-collected health Data (RECORD) statement. PloS Med. (2015) 12:e1001885. doi: 10.1371/journal.pmed.1001885 26440803 PMC4595218

[19] International statistical classification of diseases and related health problems 10th revision (2022). Available online at: https://icd.who.int/browse10/2019/en.

[20] Morken IS Viddal KR von Soest T Wichstrøm L. Explaining the female preponderance in adolescent depression-A four-wave cohort study. Res Child Adolesc Psychopathol. (2023) 51:859–69. doi: 10.1007/s10802-023-01031-6 PMC10195739 36738407

[21] Zygo M Pawłowska B Potembska E Dreher P Kapka-Skrzypczak L. Prevalence and selected risk factors of suicidal ideation, suicidal tendencies and suicide attempts in young people aged 13–19 years. Ann Agric Environ Med. (2019) 26:329–36. doi: 10.26444/aaem/93817 31232067

[22] Goldman-Mellor S Allen K Kaplan MS. Rural/urban disparities in adolescent nonfatal suicidal ideation and suicide attempt: A population-based study. Suicide Life Threat Behav. (2018) 48:709–19. doi: 10.1111/sltb.12390 28940747

[23] Salt E Wiggins AT Cerel J Hall CM Ellis M Cooper GL. Increased rates of suicide ideation and attempts in rural dwellers following the SARS-CoV-2 pandemic. J Rural Health. (2023) 39:30–8. doi: 10.1111/jrh.12686 PMC9349837 35708462

[24] Hermosillo-de-la-Torre AE Arteaga-de-Luna SM Acevedo-Rojas DL Juárez-Loya A Jiménez-Tapia JA Pedroza-Cabrera FJ. Psychosocial Correlates of Suicidal Behavior among Adolescents under Confinement Due to the COVID-19 Pandemic in Aguascalientes, Mexico: A Cross-Sectional Population Survey. Int J Environ Res Public Health. (2021) 18:4977. doi: 10.3390/ijerph18094977 34067094 PMC8124170

[25] Ong MS Lakoma M Gees Bhosrekar S Hickok J McLean L Murphy M. Risk factors for suicide attempt in children, adolescents, and young adults hospitalized for mental health disorders. Child Adolesc Ment Health. (2021) 26(2):134–42. doi: 10.1111/camh.12400 32569425

[26] Chen H Li W Cao X Liu P Liu J Chen X. The association between suicide attempts, anxiety, and childhood maltreatment among adolescents and young adults with first depressive episodes. Front Psychiatry. (2021) 12:745470. doi: 10.3389/fpsyt.2021.745470 34975565 PMC8718918

[27] Barbeito S Vega P Sánchez-Gutiérrez T Becerra JA González-Pinto A Calvo A. A systematic review of suicide and suicide attempts in adolescents with psychotic disorders. Schizophr Res. (2021) :235:80–90. doi: 10.1016/j.schres.2021.07.029 34332428

[28] Gmitrowicz A Szymczak W Kotlicka-Antczak M. Suicidal ideation and suicide attempt in Polish adolescents: Is it a suicidal process? Int J Adolesc Med Health. (2003) 15:113–24. doi: 10.1515/IJAMH.2003.15.2.113 12955813

[29] Koppen A Thoonen IMJ Hunault CC van Velzen AG de Lange DW Rietjens SJ. Significant increase in deliberate self-poisonings among adolescents during the second year of the COVID-19 pandemic. J Adolesc Health. (2023) 73:319–24. doi: 10.1016/j.jadohealth.2023.02.041 PMC10154158 37140519

[30] Marzec I Zabłocka K Stachurski J. Suicide attempts in children and adolescents – risk factors, methods and management of suicidal patient. Pediatr Pol. (2021) 96:190–7. doi: 10.5114/POLP.2021.108226

[31] Glenn CR Kleiman EM Kellerman J Pollak O Cha CB Esposito EC. Annual Research Review: A meta-analytic review of worldwide suicide rates in adolescents. J Child Psychol Psychiatry. (2020) 61:294–308. 31373003 10.1111/jcpp.13106

[32] Pikala M Burzyńska M. The burden of suicide mortality in Poland: A 20-year register-based study (2000–2019). Int J Public Health. (2023) 68:1605621. doi: 10.3389/ijph.2023.1605621 36816833 PMC9931732

[33] Bruns N Willemsen L Stang A Kowall B Holtkamp K Kamp O. Pediatric ICU admissions after adolescent suicide attempts during the pandemic. Pediatrics. (2022) 150(2):e2021055973. doi: 10.1542/peds.2021-055973 35534988

[34] Naczelna Izba Lekarska. Informacje statystyczne (2024). Available online at: https://nil.org.pl/rejestry/centralny-rejestr-lekarzy/informacje-statystyczne.

[35] Świat przychodni (2024). Available online at: https://swiatprzychodni.pl/specjalnosci/psychiatra/.

[36] Ministerstwo zdrowia (2024). Available online at: https://www.gov.pl/web/zdrowie.

[37] Agboola OP Nia HA Dodo YA. Strengthening resilient built environments through human social capital: A path to post-COVID-19 recovery. Urban Sci. (2023) 7:114. doi: 10.3390/urbansci7040114

[38] Agboola OP Rasidi MH Said I. Adolescents’ Sense of community and involvement in playground activities: panacea to ameliorate social vices and delinquencies. Int J Built Environ Sustainability. (2017) 4(2):81–92. doi: 10.11113/ijbes.v4.n2.179

